# Factors related to the fatigue of relief workers in areas affected by the Great East Japan Earthquake: survey results 2.5 years after the disaster

**DOI:** 10.1186/s13030-018-0133-0

**Published:** 2018-10-12

**Authors:** Noriko Setou, Takaki Fukumori, Kazuhisa Nakao, Masaharu Maeda

**Affiliations:** 1 Fukushima Medical University, 1 Hikarigaoka, Fukushima City, Fukushima 960-1295 Japan; 2 Tokushima University, 1-1 Minamijosanjima-cho, Tokushima City, Tokushima 770-8502 Japan; 3 Konan Women’s University, 6-2-23, Morikita-machi, Higashinada-ku, Kobe-shi, Hyogo-ken 658-0001 Japan

**Keywords:** Relief worker, Fatigue, Loss, Distress, The Great East Japan Earthquake

## Abstract

**Background:**

After the Great East Japan Earthquake (March 11, 2011), the fatigue of relief workers became a major problem in affected areas. In the present study, we conducted a questionnaire survey 2.5 years post-disaster identifying factors related to the fatigue of relief workers.

**Methods:**

This survey was cross-sectional and participants (*N* = 119) were relief workers living in affected areas. We used a self-administered questionnaire which included participants’ current problems, sources of strong feeling of loss, psychological distress and compassion fatigue. Based on answers (Yes/No) to the fatigue item, we created 2 groups; a Fatigue-group and a Non-fatigue group. We employed bivariate analysis on items with significant differences between the two groups and entered them into a multivariable logistic regression model.

**Results:**

Fifty-seven (48%) reported that they were “very tired” and were assigned to the Fatigue group. The total score of the 6-item Kessler Psychological Distress Scale (K6) and each subscale score (burnout, secondary trauma, and compassion satisfaction) of the Professional Quality of Life measure (Pro-QOL) in the Fatigue group were significantly higher than those in the Non-fatigue group. There were significant differences between the two groups for 11 items relating to current problems and sources of strong feelings of loss, and the following items were extracted as factors related to the fatigue of relief workers: loss of trust in others (adjusted OR, 10.03: 95%CI, 2.30–43.79), no confidence to continue work (adjusted OR, 6.27: 95%CI, 1.72–22.83), loss of important person(s) (adjusted OR, 5.58: 95%CI, 2.05–15.19), and sleep disturbance (adjusted OR, 5.14: 95%CI, 1.93–13.67).

**Conclusion:**

Many relief workers who reported fatigue had experienced various losses and current problems. Adequate consideration and care systems for local relief workers with fatigue should be given for a long-period after a disaster and it is important for the workers themselves to continue accepting support from others and maintaining self-care habits.

## Background

Disasters have a huge impact not only on the people directly affected, but also on disaster relief workers. It is known that relief workers suffer from particular stress. Because of a strong sense of professional mission, it is difficult for them to pay attention to their own health issues, and they tend to put off taking care of themselves.

Especially after large-scale disasters, relief work in the affected areas continues over a long period, and the stress of relief workers changes as time goes on. For example, early responders, such as members of the Defense Forces, firefighters, and rescue workers including members of the critical care team often experience Critical Incident Stress. There have been some useful studies about the PTSD (post-traumatic stress disorder) and psychological distress of relief workers in the acute phase. These suggest that relief workers may suffer from PTSD symptoms that include sleep disturbances, nightmares, and hyper-arousal as well as psychological stresses such as feelings of helplessness, anxiety, and depression [1–5].

In the medium- to long-term period after a disaster, many outside relief workers withdraw, and restoration support is mainly offered by local relief workers who live in the area. Particularly in large-scale natural disasters, some of the relief workers are disaster victims themselves, meaning they provide long-term support while coping with their own loss and trauma at the same time. Roughly 6 months after the Great East Japan Earthquake, issues of fatigue and absence from work became major problems in the affected areas. Sakuma investigated 1294 relief and reconstruction workers 14 months after the Great East Japan Earthquake, and the results revealed a high prevalence of probable PTSD (6.6%), probable depression (14.3%), and general psychological distress (14.5%) [6].We also surveyed 156 relief workers 1.5 years into the post-disaster period and found that many of them had been victims of the disaster, with 56% reporting that they had been “very tired” [7].

Figley, a specialist in trauma care, emphasized the risk of secondary trauma in relief workers who provide trauma care in tragic situations such as a disaster, and proposed a conceptual model of compassion fatigue [8]. He defined compassion fatigue as extreme physical and mental fatigue and exhaustion as a result of helping others, and it causes extremely serious burnout.

To maintain the work of supporting affected people in the medium- and long-term period after a disaster, it is very important to prevent burnout in relief workers. At the time of the Great East Japan Earthquake, many local relief workers themselves were disaster victims, therefore, it is probable that their burnout arose not only from their relief work but also from their own sense of loss and problems. We’ve now conducted a survey 2.5 years after the Great East Japan Earthquake of relief workers who lived in disaster-stricken areas, in order to identify the personal stressors related to their fatigue.

## Methods

### Survey and ethical considerations

A cross-sectional survey was conducted from September 2013 through December 2013 (i.e., 2.5 years after the Great East Japan Earthquake). The questionnaire was directly mailed or distributed to relief workers with the assistance of local governments and professional organizations (e.g., Japan Care Manager Association and Japan Pharmaceutical Association).

The rights of participants were protected based on the ethical guidelines of the Declaration of Helsinki. The following ethical considerations were explicitly stated in the survey: responses are anonymous so that respondents cannot be identified; personal information will be strictly protected; and participation in the study is voluntary. This study protocol was approved by the Research Ethics Committee of Konan Women’s University in October 2012.

### Participants

The participants consisted of 143 local relief workers from coastal communities of Iwate and Miyagi prefectures, areas which experienced devastating damage from the Great East Japan Earthquake. All participants were directly involved in supporting disaster victims and came from the fields of health care, consultation services, home care services, and so on. Volunteer workers were excluded. Of the 143 local relief workers, 119 participants with no missing values became the subjects of analysis.

### Measures

The questionnaire included a total of 62 question items as follows:

#### Personal data

Five items of basic demographic characteristics: gender, age, marital status, occupation, and living arrangements (living alone or with family members).

Eleven items requiring a “Yes” or “No” response regarding problems the person currently has: 1) I’m very tired (= Fatigue), 2) I don’t exercise enough (= Lack of exercise), 3) I don’t have enough sleep or my sleep pattern is not normal (= Sleep disturbance), 4) I’m not eating properly (= Dietary problem), 5) I don’t have enough time to spend with my family and friends (= Lack of time with family and friends); 6) I cannot rely on anyone (= No one to rely on), 7) I hardly laugh any more (=Less laughing), 8) I’m working much too hard (= Working too hard), 9) I feel guilty when I rest or take a day off (= Guilt over taking a break), 10) I prioritize the needs of disaster victims over my own needs (= Priority to others’ needs), and 11) I lack the confidence to continue in this work (= No confidence to continue work). These items of current problems were prepared by the authors based on common complaints made by relief workers at the disaster site.

Regarding the “sense of loss”, the researchers were aware it had been 2 and a half years since the disaster and they focused the questions on current sense of loss, that is, at the time of the survey, rather than what they had actually lost. These were: 1) Family, relatives and close friends, 2) Home and possessions, 3) Job, 4) Own health, 5) Family member’s health, 6) Community, 7) Landscape and scenery, 8) Sense of safety, 9) Trust in others, and 10) Hope for the future. These 10 items required a “Yes” or “No” response.

Also, at the end of the questionnaire we asked for a freely written description of the difficulties they felt regarding continuing their work.

#### K6 and Pro-QOL

We used the Japanese version of the 6-item Kessler Psychological Distress Scale (K6) [9] and the 5th version of the 30-item Professional Quality of Life measure (Pro-QOL) to examine the relation of “Fatigue” to psychological distress and compassion fatigue. If there were any relation, it would suggest to some degree that “Fatigue” is a state of strong psychological distress or burnout.

The K6 is the Kessler Psychological Distress Scale [9] in Japanese [10] to measure general psychological distress and includes symptoms of depression and anxiety in the previous 30 days. Responses are on a five-point scale, ranging from “not at all” [0] to “all the time” [4]. Total scores range from 0 to 24, with 13 points or higher indicating a high risk of mood and anxiety disorder.

The Pro-QOL [11, 12] is a scale developed by Stamm et al. (2010) to measure the compassion satisfaction and compassion fatigue of relief workers on the basis of the compassion fatigue model by Figley. The 5th version, translated into Japanese by Toyomi Goto, was used in this survey. The scale consists of 10 items, each with three subscales; “compassion satisfaction”, “burnout”, and “secondary trauma” (30 items in total). The frequency of each item experienced in the previous 30 days was placed on a scale of 1 to 5 (from “never” [1] to “very often” [5]). The t-score is used to standardize the score to make the median score (t-score of 50 points) represent the mean, which enables identification of high-risk individuals in each subscale. The t-score is a value obtained by multiplying the z-score (a value obtained by subtracting the average value from the raw score and dividing the value by the standard deviation) by 10, then adding 50. The present study used the t-score cutoff values recommended by Stamm et al. to determine high-risk participants. Based on this, 40 points or lower for compassion satisfaction, 57 points or higher for burnout, and 57 points or higher for secondary trauma indicated high risk [13] (see Appendix).

### Statistical analysis

Participants who responded “Yes” to “Fatigue” were classified into a Fatigue Group and those who responded “No” as a Non-fatigue Group.

First, we confirmed the normality of the total score of the K6. Then, we conducted a t-test comparing the Fatigue group with the Non-fatigue Group to confirm the relation of “Fatigue” to the K6 and each subscale of the Pro-QOL (compassion satisfaction, burnout, and secondary trauma).

Second, we conducted a chi-square test and calculated the significance probability comparing the two groups over a total of 25 items including 4 demographic characteristics, 11 current problems, and 10 sources of strong feelings of loss in order to examine the relation between fatigue and current personal information. We eliminated the job category in the demographic characteristics from our statistical analysis since all of the respondents directly supported disaster victims.

Then, we performed independent chi-squared tests between the items with a *p*-value < 0.2 and “fatigue” items used as independent variables before logistic regression analysis to analyze the factors related to fatigue. In addition, logistic regression analysis with a sequential method increasing variables was performed, and then an odds ratio (OR) was calculated. All statistical analyses were performed using the Statistical Package for Social Science (SPSS) version 25.0 for Windows. Statistical significance was set at 0.05.

## Results

The number of participants was 119, including 26 public health nurses and clinical nurses; 38 other professionals such as care managers, pharmacists, and psychologists; 23 civil servants; 14 home care and reconstruction supporters; and 18 staff members from NPOs involved in supporting disaster victims. All participants were directly engaged in supporting victims and lived in the disaster-stricken areas.

For “Fatigue”, 48% (*N* = 57) of the participants answered “Yes”. Table 1 shows the chi-square test results of 26 items for the basic demographic characteristics, current problems, and source of strong sense of loss between the Fatigue Group and Non-fatigue Group. Significant differences were seen in 11 items: “Female”, “Sleep disturbance”, “Dietary problem”, “Guilt over taking a break”, “No confidence to continue work”, “Loss of important person(s)”, “Loss of job”, “Loss of one’s own health”, “Loss of the health of a family member”, “Loss of community”, and “Loss of trust in others”. The mean number of items with “Yes” for “current problems” was 3.2 and for “source of strong sense of loss” was 2.6 per participant.

**Table 1:** Demographic characteristics, current problems, sources of strong feeling of loss

	Participants (Total = 119)		Fatigue		Non-fatigue		df	χ²	φ	*P*-value
	*N*	%	*N*	%	*N*	%				
Demographic characteristics										
Female Sex	74	62.2	41	55.4	33	44.6	1	4.4	0.19	**0.04** ^**a**^
Age <40	46	33.6	19	41.3	27	58.7	3	1.3		0.26
Single	20	15.8	9	45.0	11	55.0	1	1.0		0.60
Married	78	65.5	36	45.2	42	54.8	1	2.0		0.37
Current problems										
Fatigue	57	47.9	-	-	-	-		-	-	-
Lack of exercise	80	67.2	43	53.2	37	46.3	1	3.3	0.17	**0.08**
Sleep disturbance	38	31.9	28	73.7	10	26.3	1	14.8	0.35	***p*** <0.01**
Dietary problem	27	22.7	19	70.4	8	29.6	1	7.1	0.24	***p*** <0.01**
Lack of time with family and friends	26	21.8	14	53.8	12	46.2	1	0.5		0.51
No one to rely on	38	31.9	20	52.6	18	47.4	1	0.5		0.56
Less laughing	22	18.5	15	68.2	7	31.8	1	4.4	0.19	**0.06**
Working too hard	23	19.3	14	60.9	9	39.1	1	1.9		0.25
Guilt over taking a break	25	21.0	17	68.0	8	32.0	1	5.1	0.21	**0.03** ^**a**^
Priority to others’ needs	27	22.7	16	59.3	11	40.7	1	1.8	0.12	**0.20**
No confidence to continue work	21	17.6	17	81.0	4	19.0	1	11.2	0.31	***p*** <0.01**
Sources of strong feeling of loss										
Family, relatives, and close friends	33	27.7	23	69.7	10	30.3	1	8.7	0.27	***p*** <0.01**
Home and possessions	25	21	15	60.0	10	40.0	1	1.8	0.13	**0.19**
Job	4	3.4	4	100	0	0	1	4.5	0.19	**0.05** ^**a**^
Personal health	12	10.1	11	91.7	1	8.3	1	10.2	0.28	***p*** <0.01**
Family member’s health	14	11.8	12	85.7	2	14.3	1	9.1	0.27	***p*** <0.01**
Community	27	22.7	18	66.7	9	33.3	1	4.9	0.20	**0.03** ^**a**^
Landscape and scenery	66	55.5	33	50.0	33	50.0	1	0.26		0.71
Sense of safety	52	43.7	30	57.7	22	42.3	1	3.5	0.17	**0.07**
Trust in others	17	14.3	14	82.4	3	17.6	1	9.4	0.28	***p*** <0.01**
Hope for the future	23	19.3	14	60.9	9	39.1	1	1.9	0.13	0.245

^a^The bold items are final explanatory variables for multiple logistic analysis

*p<0.05; **p<0.01

In the free description of feelings of difficulty regarding continuing work, there were several answers, such as “I cannot see the direction of restoration support.” “I sense a difference in enthusiasm between disaster support units and non-disaster support units” and, “I feel like disaster relief workers are gradually being left behind”.

We examined the differences of all K6 scores (raw data) and each t-score of the Pro-QOL subscale items (compassion satisfaction, burnout, and secondary trauma) between the Fatigue Group and the Non-fatigue Group. As shown in Table 2, all items showed significant differences.

**Table 2:** K6 and Pro-QOL subscale scores

	Fatigue *N* = 57	Non-fatigue *N* = 62	Statistic
	Mean ± SD (Number of high risk)	Mean ± SD (Number of high risk)	*p*-value
K6*			
Total score	143.7 ± 5.0 (33)	9.7 ± 3.5 (12)	*p* < 0.01
Pro-QOL**			
Compassion Satisfaction	47.7 ± 9.5 (10)	52.1 ± 10.0 (7)	*p* < 0.05
Burnout	54.0 ± 9.5 (24)	46.3 ± 9.1 (6)	*p* < 0.01
Secondary trauma	54.0 ± 11.2 (24)	46.3 ± 7.0 (5)	*p* < 0.01

*K6 The 6-item Kessler psychological distress scale

**Pro-QOL The 5th Japanese version of professional quality of life measure

Each score of the Pro-QOL subscales is converted to t-score

In conducting the logistic regression analysis, we performed a chi-square test of independence on 16 items, which consisted of 11 items that showed significant difference to the chi-square test plus 5 items with a *p*-value < 0.2, “Lack of exercise”, “Less laughing”, “Priority to others’ needs”, “Loss of home and possessions”, and “Loss of sense of safety”, and no item exhibited a high correlation (φ>0.35) with fatigue. Then, a logistic regression analysis was performed on 16 items as independent variables. As a result, the following 4 items were extracted as fatigue-related factors: “Sleep disturbance”, “No confidence to continue work”, “Loss of important person(s)”, and “Loss of trust in others”. Each odds ratio and 95% confidence interval is shown in Table 3.The result of the Hosmer-Lemeshow test indicated a good fit (*p* = 0.897), and the discriminant predictive value was 75.4%.

**Table 3:** Fatigue-related factorelated factors of disaster relief workers

*N* = 119	β	SE*	OR**	95% CI	*p*-value
Sleep disturbance	1.64	0.50	5.14	1.93–13.67	.001
No confidence to continue work	1.84	0.66	6.27	1.72–22.83	.005
Loss of family and/or close friend(s)	1.72	0.51	5.58	2.05–15.19	.001
Loss of trust in others.	2.31	0.75	10.03	2.30–43.79	.002

*SE Standard error, **OR Odds ratio

## Discussion

This survey focused on factors related to the fatigue of relief workers over the medium- and long-term period after the Great East Japan Earthquake. The main findings can be summarized as follows:

The results revealed that 48% of the participants were experiencing strong fatigue (= Fatigue Group) 2.5 years after the disaster, and this high percentage of relief workers with feelings of fatigue has continued. The Fatigue Group showed significantly high values in the total score of the K6 and t-score of the Pro-QOL’s subscale compared to the Non-fatigue Group. Maeda [14] conducted a survey with 168 public employees working in the affected coastal area between 24 and 30 months after the Great East Japan Earthquake and reported that the percentage of people with current depression was as high as 17.9%, which indicated the seriousness of their mental state. Similarly, in this study, participants categorized in the Fatigue Group were shown to be at high risk in terms of both psychological distress and compassion fatigue.

Significant differences in the chi-square test were shown between the Fatigue Group and Non-fatigue Group in 11 items. These were “Gender”, “Sleep disturbance”, “Dietary problems”, “Guilt over taking a break”, “No confidence to continue work”, “Loss of important person(s)”, “Loss of job”, “Loss of one’s own health”, “Loss of the health of family member”, “Loss of community”, and “Loss of trust in others”. Six of these items related to a strong feeling of loss, suggesting that not only the influence of trauma but also the influence of loss continue for a long time after a disaster.

Hobfall et al. emphasized that distress following a disaster often arises after a victim’s resources were lost or damaged [15] and stated that in order to support the psychological recovery of affected people it is necessary to focus on the status and inter-relationships of each of three resources: “the community they live in”, “extrinsic resources” such as interpersonal relationships, and “intrinsic resources” such as the individual’s emotion and cognition [16]. Many communities in the coastal areas stricken by the Great East Japan Earthquake had not been fully rebuilt even 2.5 years after the disaster. The results of the present study indicate that relief workers who complain of fatigue may not have recovered their extrinsic and intrinsic resources or that they may have continued to experience deep feelings of loss even if they had recovered the material aspects. It is possible that the relief workers have been supporting victims suffering from trauma while in that state. Figley, who originated the conceptual model of compassion fatigue, stated “there is a cost to caring” [17] and emphasized that if a relief worker who hasn’t been coping with their fatigue and distress continues to support affected people with trauma, their personal lives will be at risk over time and they are likely to experience burnout [8, 17]. The participants with fatigue in this study were considered to be at high risk of burnout because many were currently experiencing multiple problems.

According to the logistic regression analysis, the following items were linked to fatigue: “Sleep disturbance”, “No confidence to continue work”, “Loss of important person(s)”, and “Loss of trust in others”. “Loss of trust in others”, in particular, showed the highest odds ratio. Although it is not possible to determine the reason from this survey alone, there have been situations where relief workers encounter conflict in interpersonal relationships, such as being insulted and verbally abused by affected people as well as being embroiled in quarrels among them [18]. Another possible factor is the enthusiasm gap among relief workers. In this study there were several comments in the free-description section of the questionnaire, stating “I sense a difference in enthusiasm between disaster support units and non-disaster support units” and “I feel like disaster relief workers are gradually being left behind”. The fact that employment in victim support services is often on a year-to-year basis may also be a factor in the unstable relationships in the workplace. Indeed, we often hear these opinions voiced in disaster-stricken areas and we think it is possible that the lack of recognition of the efforts of those relief workers by the people around them may have become one of the major causes of distress.

“Confidence to continue work” is known as a factor that affects “burnout” and “work engagement (a positive and fulfilling psychological state related to work)”. These are defined by two factors: One is related to work, such as sense of control of work and remuneration, and the other is personal factors, such as positive self-evaluation of work and self-efficacy [19, 20]^.^ “No confidence to continue work” is considered to be the latter factor. These intrinsic problems are directly connected to burnout, and should be taken into consideration much more.

“Loss of important person(s)” was also revealed as a fatigue-related factor. As Figley pointed out, disaster relief workers are prone to suffer from secondary trauma and may rekindle their own trauma when supporting victims [8]. In order to prevent burnout in those relief workers who have experienced the loss of a close person(s), it is very important that they give care to their own experiences of loss and receive professional supervision in their work.

In recent years, “resilience” has become an essential and important element for the psychological recovery of disaster victims. Resilience can be described as “the strength to continue living a stable life despite unresolvable circumstances”, and a connection and close ties to other people are absolutely imperative for increasing resilience [21].

It is important to consider the “resilience of local relief workers” if they are to continue providing long-term support following a disaster. To that end, people inside and outside of disaster areas need to acknowledge that “Relief workers also need support for the long-term period after a disaster”. Adequate consideration and care systems for local relief workers should be given so that they can avoid psychologically being left behind. Furthermore, efforts to prevent secondary mental injury and promote a “connection with people” are required so that relief workers themselves can recover from their various loss and trauma experiences in their human relationships.

Moreover, local relief workers themselves need to pay more attention to their own health, lifestyle including diet and sleep, inner feelings, and symptoms of distress so as to improve their own coping abilities as well as self-reliance. In particular, sleep disturbance is highly related to fatigue, so it is very important that relief workers do not hesitate to ask for help and have support from others if they continue to feel fatigue that affects their lifestyle. It is also important for them to maintain the habit of taking care of themselves. Since there are cultural norms that endurance is a virtue and a resistance to seeking support in Japan [22], psychological education for Japanese relief workers is especially recommended.

## Study limitations

The limitations of this research are described in the following three points:

The first point concerns the validity of the fatigue item. In this study, we did not conduct interviews but used questionnaires. Also, we assumed that its validity is, to some extent, relevant to burnout and compassion fatigue, with these concepts being closely related. However, the validity of fatigue measured with Yes/No responses is not clear, and it has not been examined in previous studies.

The second point concerns the explanatory variables and items used. The items “current problems” and “sources of strong feeling of loss” were created by us and are not from standardized scales. Because the Japanese version of the 5th Pro-QOL has not yet been standardized, we used the overseas standard score (Appendix) used by Stamm in this study.

The third point concerns the number of participants. Given the total number of relief workers in the affected area, our sample was very small, thus this survey might not reflect the circumstances of relief workers as a whole.

However, we believe this survey shows some aspects of the mental health of relief workers 2.5 years after the Great East Japan Earthquake, since we limited the subjects to coastal areas and conducted surveys of participants who were directly involved in continuous support of disaster victims, and many items of the questionnaire accurately reflect the voices of relief workers in the affected areas. We hope to follow up changes in fatigue and distress to create a support system for local relief workers in the future.

## Conclusion

The fatigue of relief workers has continued for 2.5 years after the Great East Japan Earthquake. Our findings from scores generated by K6 and the subscales of Pro-QOL revealed relief workers with fatigue had a high risk of burnout and compassion fatigue. Also, fatigue was considered to be an index of the post-disaster mental health status of relief workers. The following items were extracted as fatigue-related factors: “Sleep disturbance”, “No confidence to continue work”, “Loss of important person(s)”, and “Loss of trust in others”. It is imperative to provide long-term support for local relief workers with fatigue after a disaster.

## Appendix

**Table 4:** T-score cut-off point of Pro-QOL ver.5 (Original)

	High	Moderate	Low
Compassion satisfaction	≧57	44 < 56>	≦40^a^
Burnout	≧57^a^	44 < 56>	< 43
Secondary trauma	≧57^a^	44 < 56>	< 43

^a^The high risk score is 40 points or lower for compassion satisfaction, 57 points or higher for burnout, and 57 points or higher for secondary trauma

## Abbreviations

95%CI: 95% confidence interval
K6: The 6-item Kessler Psychological Distress Scale
OR: Odds ratio
Pro-QOL: The 30-item Professional Quality of Life measure
PTSD: Post-traumatic stress disorder
SE: Standard error
SPSS: Statistical Package for Social Science
